# Ankle-Brachial Index Is a Powerful Predictor of Renal Outcome and Cardiovascular Events in Patients with Chronic Kidney Disease

**DOI:** 10.1100/2012/238494

**Published:** 2012-01-04

**Authors:** Fu-An Chen, Chih-Yu Yang, Wu-Chang Yang, Jinn-Yang Chen, Yee-Yung Ng, Szu-Yuan Li, Wen-Sheng Liu, Shiao-Ti Cheng, Yu-Jen Wang, Chih-Ching Lin

**Affiliations:** ^1^School of Medicine, National Yang-Ming University, No. 155, Section 2, Linong Street, Taipei, Taiwan; ^2^Division of Nephrology, Department of Internal Medicine, National Yang-Ming University Hospital, No. 152, Xinmin Road, Yilan, Taiwan; ^3^Division of Nephrology, Department of Internal Medicine, Taipei Veterans General Hospital, No. 201, Section 2, Shih-Pai Road, Taipei, Taiwan; ^4^Department of Nursing, Taipei Veterans General Hospital, No. 201, Section 2, Shih-Pai Road, Taipei, Taiwan

## Abstract

Ankle-brachial index (ABI) is an accurate tool to diagnose peripheral arterial disease. The aim of this study was to evaluate whether ABI is also a good predictor of renal outcome and cardiovascular events in patients with chronic kidney disease (CKD). We enrolled 436 patients with stage 3–5 CKD who had not been undergoing dialysis. Patients were stratified into two groups according to the ABI value with a cut point of 0.9. The composite renal outcome, including doubling of serum creatinine level and commencement of dialysis, and the incidence of cardiovascular events were compared between the two groups. After a median follow-up period of 13 months, the lower ABI group had a poorer composite renal outcome (OR = 2.719, *P* = 0.015) and a higher incidence of cardiovascular events (OR = 3.260, *P* = 0.001). Our findings illustrated that ABI is a powerful predictor of cardiovascular events and renal outcome in patients with CKD.

## 1. Introduction

Chronic kidney disease (CKD) is a growing health concern worldwide. According to several epidemiologic studies, the prevalence of CKD ranges from 11.9% to 16.2% in different countries [1–4]. CKD is associated with high cardiovascular mortality [5], which is the leading cause of mortality in many developed countries. Furthermore, many patients with CKD develop end-stage renal disease (ESRD), which is a tremendous burden on health care systems [6]. Thus, simple and effective methods are required to identify such high-risk patients and to prevent them from developing cardiovascular events or ESRD [7].

The ankle-brachial index (ABI) is a good tool to evaluate systemic atherosclerosis [8] and the outcome of cardiovascular disease. An ABI less than 0.9 is highly sensitive and specific for the diagnosis of peripheral arterial occlusive disease (PAOD) [9], which is a prominent risk factor for ischemic heart disease and cardiovascular mortality [10]. The aim of this study was to evaluate whether low ABI is a good predictive factor for determining renal outcome and cardiovascular events in patients with CKD.

## 2. Material and Methods

### 2.1. Study Population

This prospective observational study was conducted between October 2008 and January 2010 in a tertiary referral center. We enrolled patients older than 18 years of age with a GFR of less than 60 ml/min/1.73 m^2^, as calculated by the modification of diet in renal disease equation. Patients undergoing renal replacement therapy or those who had undergone renal transplantation or bilateral leg amputation were excluded from the study. The study protocol was approved by the Institutional Review Board of our hospital. All participants provided written informed consent.

### 2.2. ABI Measurement

After enrollment, the subjects' ABI was measured at the outpatient clinic. Measurements of ABI were obtained using an ABI-form device (VP1000; Colin, Komaki, Japan), which automatically and simultaneously measures blood pressure in both the arms and the ankles by using an oscillometric method. The ABI was calculated as the ratio of the systolic blood pressure of the ankle to that of the arm. The lowest ABI obtained from either leg was taken as the patient's ABI value. The ABI measurement was done once in each patient.

### 2.3. Collection of Demographic and Laboratory Data

Demographic data, including age, gender, diabetes mellitus status, hypertension status, and use of prescribed antihypertensive agents, were obtained from the patients' medical records. A patient was considered to have hypertension if the systemic blood pressure was more than 140 mmHg or diastolic blood pressure was more than 90 mmHg or if the patient was currently using antihypertensive agents. A patient was considered to have diabetes mellitus if the fasting blood sugar was more than 7 mmol/L or if the patient was using oral antidiabetic agents or insulin. Systemic blood pressure, diastolic blood pressure, pulse pressure, and body mass index (BMI) were recorded during the same visit when ABI was measured. The BMI was calculated as the ratio of the body weight in kilograms to the square of height in meters. Laboratory data, including levels of serum albumin, cholesterol, uric acid, calcium, phosphate, and hemoglobin, were measured from fasting blood samples. Urine protein/creatinine ratio (UPCR) was measured from the first morning voiding. These data were acquired within 1 month of the measurement of ABI.

### 2.4. Study Followup and Endpoints

All participants received regular followup in our nephrology clinic. Serum creatinine levels were measured every 3 months to determine the decline in renal function. The composite renal outcome was defined as doubling of serum creatinine or commencement of renal replacement therapy (hemodialysis, peritoneal dialysis, or renal transplantation). Another study outcome was the occurrence of cardiovascular events, defined as mortality or hospitalization because of cerebrovascular events, myocardial infarction, congested heart failure, or peripheral vascular diseases.

### 2.5. Statistical Analysis

All statistical analyses were performed using SPSS 16.0 for Windows (SPSS Inc., Chicago, Il, USA). Data were expressed as mean ± standard deviation or as a percentage. For analysis of the determinants of low ABI, a chi-square test was used for categorical variables, and an independent *t*-test was used for continuous variables. Multivariate forward logistic regression analysis was applied to identify the independent determinant factors of low ABI. To determine the influence of ABI on renal and cardiovascular outcome, we used the Kaplan-Meier analysis. Cox regression model was used to evaluate the effect of presumed risk factors on renal and cardiovascular outcome. Variables with a *P* value less than 0.1 in univariate Cox regression analysis were included in a multivariate Cox regression analysis.

## 3. Results

In this study, we enrolled 436 patients. The baseline characteristics of the study population are shown in Table 1. The mean age was 73.4 ± 10.5 years, 36.9% of the patients had diabetes mellitus, and 87.4% of the patients were hypertensive. The prevalence of low ABI was 12.4%, and the mean GFR was 28.0 ± 13.0 ml/min/1.73 m^2^. Patients with ABI < 0.9 tended to be older and have a higher prevalence of diabetes and hypertension, higher systolic blood pressure and pulse pressure, lower serum albumin level, higher UPCR, and a higher prevalence of calcium channel blocker usage. Multivariate logistic regression analysis was performed to analyze the determinants of low ABI in our subjects. The results are listed in Table 2. ABI < 0.9 was associated with old age (OR = 2.020, *P* = 0.001), low GFR (OR = 0.727, *P* = 0.017), diabetes mellitus (OR = 2.360, *P* = 0.006), and high pulse pressure (OR = 1.365, *P* = 0.002).

**Figure 1:**
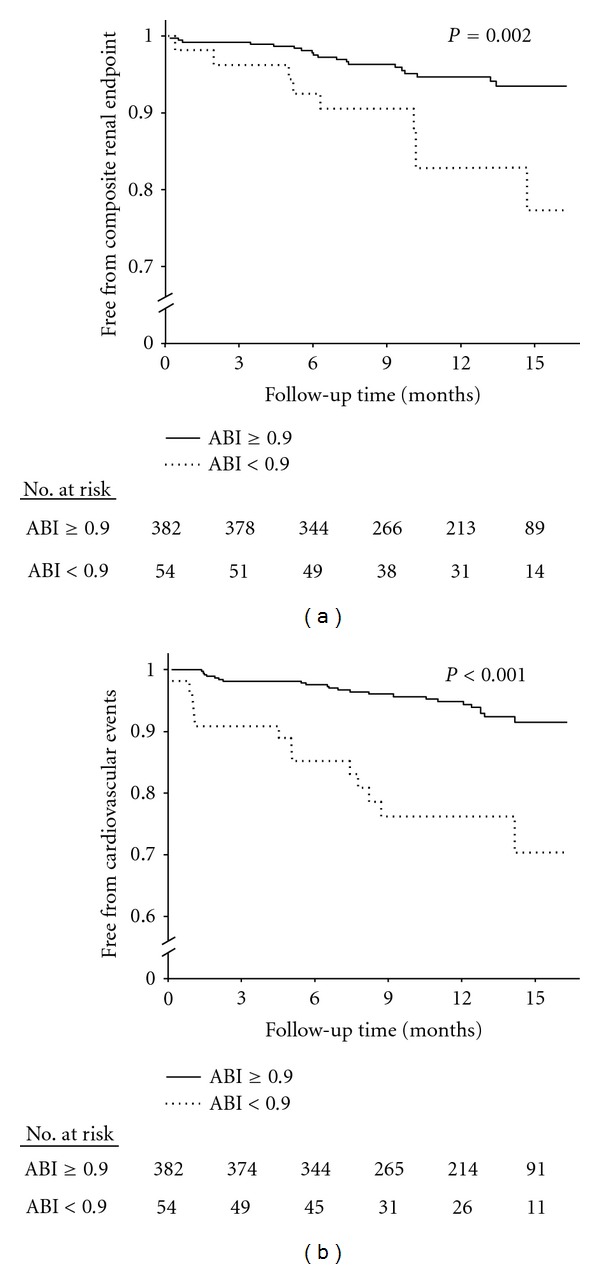
The relationship between ABI and composite renal endpoint and cardiovascular events. Kaplan-Meier's curves with log-rank tests of (a) freedom from composite renal endpoint and (b) freedom from cardiovascular events in patients with chronic kidney disease stratified according to the level of ankle-brachial index (ABI).

The median follow-up time of the study was 13 months. In low ABI group, two patients died (3.7%), where as six patients died in the ABI > 0.9 group (1.6%). Nine patients (16.7%) with ABI < 0.9 and sixteen patients (5.0%) with ABI ≥ 0.9 reached composite renal endpoint, and thirteen patients (24.1%) with ABI < 0.9 and twenty-three patients (6.0%) with ABI ≥ 0.9 developed cardiovascular events. Compared to those with ABI ≥ 0.9, the low ABI group had poorer composite renal outcome (*P* = 0.002) and higher incidence of cardiovascular events (*P* < 0.001), as revealed by the Kaplan-Meier analysis (Figure 1). In multivariate Cox regression analyses, low ABI was still an independent predictive risk factor of composite renal outcome (OR = 2.719, *P* = 0.015) (Table 3) and cardiovascular events (OR = 3.260, *P* = 0.001) (Table 4).

## 4. Discussion

ABI has been well recognized as a diagnostic index of PAOD [9] and a marker for atherosclerosis. It has also been shown to be a good prognostic tool to evaluate the risk of stroke, myocardial infarction, progression of renal failure, and cardiovascular death in normal populations [11–14]. Similarly, ABI has also been shown to be a good marker for predicting cardiovascular mortality in CKD patients irrespective of whether they are receiving renal replacement therapy [15, 16]. However, its power to predict a decline in renal function in patients with CKD has not been well studied. Our analysis revealed that low ABI is associated with a rapid decline in renal function. In a multivariate Cox regression analysis, patients with lower ABI had a 2.72-fold higher risk for rapid deterioration of renal function. Renal failure is a well-known risk factor for systemic atherosclerosis, which can cause various cardiovascular diseases. However, recent studies suggest that atherosclerosis, in turn, may exacerbate renal failure. O'Hare et al. reported that a lower ABI value was related to an increase in serum creatinine level over time in a normal population [14]. Feringa et al. also demonstrated that every 0.1 decrease in the ABI level was associated with a 1.43-fold increase in the risk of development of ESRD in patients with PAOD [13]. In this study, we found that ABI was an independent prognostic factor for the decline in renal function in patients with CKD, irrespective of the underlying cause of renal disease, such as diabetes or hypertension. Therefore, this finding suggests that systemic atherosclerosis may be implicated in a common pathogenic pathway in renal dysfunction.

In addition to progressive renal failure, cardiovascular diseases are a major concern in patients with CKD. Several studies have implicated poor renal function as a factor in increasing cardiovascular events and mortality [5, 17, 18]. Among CKD patients, those with low ABI have a higher rate of cardiovascular events and mortality [15, 16, 19]. Our results are consistent with this observation. Twenty-four percent of patients with low ABI developed cardiovascular events during a mean follow-up period of 13 months. This translates into a 3.3-fold higher risk for developing cardiovascular events in patients with ABI < 0.9 than in patients with ABI ≥ 0.9. Aggressive treatment may be needed to prevent cardiovascular events in this high-risk group.

Although results from our study suggest that ABI is a good indicator of renal and cardiovascular outcomes in patients with CKD stage 3–5, our study population was selected from a single center, and hence may not be fully representative of the CKD population at large. Therefore, similar studies are required to confirm the predictive power of ABI in other populations. Furthermore, in our study, we defined cardiovascular events as either hospitalization or death due to cardiovascular disease. Since hospitalization may partly reflect disease severity and has been used as a surrogate endpoint in several large clinical trials, we thought that this would be an adequate definition of cardiovascular endpoint. However, since hospital admission criteria for cardiovascular disease may differ from place to place, redefining a cardiovascular event as cardiovascular mortality may reduce the variability introduced by such differences. In our follow-up period, we observed very few cardiovascular mortalities. Therefore, extended follow-up studies are needed to determine whether defining a cardiovascular event as cardiovascular mortality might better define the association between ABI and cardiovascular events. Similarly, because of differences in age, gender, and baseline renal function in our study population, defining renal endpoint as a doubling of serum creatinine may not be as accurate as defining it as a decline in GFR. However, since all the enrolled subjects had a GFR less than 60 ml/min/1.73 m^2^, a decline in GFR of more than 4 ml/min/1.73 m^2^ per year, which is the definition for rapid decline in renal function as in K/DOQI guideline [20], was less indicative of disease severity than a doubling of serum creatinine level in the follow-up period. Thus, we believe that our definition of renal outcome is appropriate.

## 5. Conclusion

Our study shows that patients with stage 3–5 CKD who have a lower ABI have a rapid decline in renal function and a high incidence of cardiovascular events. Since ESRD and cardiovascular diseases are major risks in patients with CKD, we suggest that ABI, a noninvasive and convenient tool to diagnose PAOD and systemic atherosclerosis, be used in all these patients.

##  Authors' Contribution

Fu-An Chen contributed to the paper processing by (1) conception and design of the study and collection and analysis of data, (2) drafting the paper, and (3) final approval of the version to be published; Wu-Chang Yang by (1) analysis and interpretation of data, (2) revising the paper, and (3) final approval of the version to be published; Chih-Yu Yang by (1) analysis and interpretation of data, (2) revising the paper, and (3) final approval of the version to be published; Jinn-Yang Chen by (1) patient referral, care, and acquisition of data and (2) final approval of the version to be published; Yee-Yung Ng by (1) patient referral, care, and acquisition of data and (2) final approval of the version to be published; Szu-Yuan Li by (1) patient referral, care, and acquisition of data and (2) final approval of the version to be published; Wen-Sheng Liu by (1) acquisition of data and (2) final approval of the version to be published; Shiao-Ti Cheng by (1) collection and analysis of data and (2) final approval of the version to be published; Yu-Jen Wang by (1) collection and analysis of data and (2) final approval of the version to be published; Chih-Ching Lin by (1) conception and design of the study and analysis and interpretation of data, (2) revising the paper, and (3) final approval of the version to be published.

## Figures and Tables

**Table 1:** Baseline characteristics of study participants grouped by ABI.

Characteristic	All patients *N* = 436	ABI < 0.9 *N* = 54	ABI ≧ 0.9 *N* = 382	*P* value
Age (year)	73.4 ± 10.5	77.7 ± 7.4	72.8 ± 10.7	<0.001
Male gender (%)	77.8	85.2	76.7	0.161
Diabetes mellitus (%)	36.9	57.4	34.0	0.001
Hypertension (%)	87.4	96.3	86.1	0.035
Mean ABI	1.06 ± 0.17	0.71 ± 0.15	1.11 ± 0.09	<0.001
Systolic BP (mmHg)	144.3 ± 20.2	152.6 ± 22.5	143.1 ± 19.6	0.001
Diastolic BP (mmHg)	77.5 ± 10.8	77.2 ± 11.8	77.5 ± 10.7	0.835
Pulse pressure (mmHg)	66.8 ± 15.0	75.4 ± 17.0	65.6 ± 14.3	<0.001
BMI	24.8 ± 3.7	24.7 ± 3.5	24.8 ± 3.8	0.943
Albumin level (g/L)	41.1 ± 4.3	39.8 ± 4.4	41.3 ± 4.3	0.014
Hemoglobin level (g/L)	115.1 ± 18.4	113.7 ± 18.2	115.3 ± 18.5	0.563
Cholesterol level (mmol/L)	4.41 ± 0.96	4.29 ± 0.76	4.42 ± 0.99	0.386
Uric acid level (*μ*mol/L)	389.4 ± 104.3	413.2 ± 100.1	386.1 ± 104.6	0.074
Calcium level (mmol/L)	2.27 ± 0.12	2.25 ± 0.12	2.27 ± 0.13	0.326
Phosphate (mmol/L)	1.16 ± 0.25	1.17 ± 0.23	1.16 ± 0.26	0.654
UPCR (g/g)	1.6 ± 2.7	2.5 ± 3.5	1.5 ± 2.5	0.041
GFR (ml/min/1.73 m^2^)	28.0 ± 13.0	24.8 ± 11.7	28.4 ± 13.2	0.057
CKD stage				0.219
Stage 3	40.4	29.6	41.9	
Stage 4	40.4	46.3	39.5	
Stage 5	19.3	24.1	18.6	
Medication				
ACE-I or ARB (%)	46.1	51.9	45.3	0.365
CCB (%)	50.5	66.7	48.2	0.011
*β*-blocker (%)	17.4	22.2	16.8	0.321
Statin (%)	21.8	27.8	20.9	0.255

ABI: ankle-brachial index, BP: blood pressure, BMI: body mass index, UPCR: urine protein/creatinine ratio, GFR: glomerular filtration rate, CKD: chronic kidney disease, ACE-I: angiotensin converting enzyme inhibitor, ARB: angiotensin II receptor blocker, CCB: calcium channel blocker.

**Table 2:** Multivariate logistic regression analysis of determinant factors of low ABI in patients with chronic kidney disease.

	Hazard ratio	95% CI Lower	95% CI Upper	*P* value
Age (per 10 years)	2.020	1.351	3.021	0.001
GFR (per 10 ml/min/1.73 m^2^)	0.727	0.560	0.944	0.017
Diabetes mellitus	2.360	1.278	4.357	0.006
Pulse pressure (per 10 mmHg)	1.365	1.122	1.662	0.002

GFR: glomerular filtration rate, CI: confidence interval.

**Table 3:** Cox proportional analysis for composite renal outcome.

Parameter	Univariate		Multivariate (forward)	
	Hazard ratio	*P* value	Hazard ratio	*P* value
Age (per 10 years)	0.808 (0.591 to 1.104)	0.180		
Male versus female	0.590 (0.267 to 1.304)	0.192		
Hypertension	1.968 (0.467 to 8.296)	0.356		
Diabetes mellitus	1.893 (0.901 to 3.976)	0.092		
PAOD	3.285 (1.486 to 7.262)	0.003	2.719 (1.214 to 6.092)	0.015
GFR (per 10 ml/min/1.73 m^2^)	0.271 (0.167 to 0.441)	<0.001	0.280 (0.162 to 0.484)	<0.001
Systolic BP (per 10 mmHg)	1.241 (1.054 to 1.463)	0.010		
Diastolic BP (per 10 mmHg)	1.454 (1.053 to 2.007)	0.023		
Pulse pressure (per 10 mmHg)	1.235 (0.987 to 1.544)	0.065		
BMI (kg/m^2^)	0.989 (0.896 to 1.092)	0.833		
Albumin (per 1 g/L)	0.822 (0.772 to 0.875)	<0.001	0.890 (0.812 to 0.975)	0.013
Hb (per 1 g/L)	0.954 (0.933 to 0.976)	<0.001		
Cholesterol (per 1 mmol/L)	1.224 (0.852 to 1.758)	0.274		
Uric acid (per 1 *μ*mol/L)	1.003 (1.000 to 1.006)	0.073		
Calcium (per 1 mmol/L)	0.007 (0.001 to 0.095)	<0.001		
Phosphate (per 1 mmol/L)	4.090 (2.117 to 7.899)	<0.001		
UPCR (per 1 g/g)	1.291 (1.216 to 1.370)	<0.001	1.115 (1.010 to 1.231)	0.030

PAOD: peripheral arterial occlusive disease, GFR: glomerular filtration rate, BMI: body mass index, UPCR: urine protein/urine creatinine ratio.

**Table 4:** Cox proportional analysis for cardiovascular events.

Parameter	Univariate		Multivariate (forward)	
	Hazard ratio	*P *value	Hazard ratio	*P *value
Age (per 10 years)	2.135 (1.356 to 3.364)	0.001	2.073 (1.297 to 3.311)	0.002
Male versus female	1.191 (0.521 to 2.719)	0.679		
Hypertension	1.721 (0.528 to 5.613)	0.368		
Diabetes mellitus	1.312 (0.676 to 2.547)	0.423		
PAOD	4.402 (2.230 to 8.691)	<0.001	3.260 (1.644 to 6.463)	0.001
GFR (per 10 ml/min/1.73 m^2^)	0.920 (0.714 to 1.184)	0.516		
Systolic BP (per 10 mmHg)	1.136 (0.976 to 1.323)	0.100		
Diastolic BP (per 10 mmHg)	1.085 (0.805 to 1.463)	0.591		
Pulse pressure (per 10 mmHg)	1.209 (0.988 to 1.480)	0.065		
BMI (kg/m^2^)	1.016 (0.930 to 1.109)	0.726		
Albumin (per 1 g/L)	0.922 (0.868 to 0.980)	0.009		
Hb (per 1 g/L)	1.005 (0.987 to 1.023)	0.607		
Cholesterol (per 1 mmol/L)	1.098 (0.759 to 1.589)	0.620		
Uric acid (per 1 *μ*mol/L)	1.004 (1.002 to 1.007)	0.001	1.004 (1.002 to 1.007)	0.002
Calcium (per 1 mmol/L)	2.669 (0.191 to 37.29)	0.466		
Phosphate (per 1 mmol/L)	1.400 (0.451 to 4.349)	0.561		
UPCR (per 1 g/g)	1.095 (1.010 to 1.186)	0.027		

PAOD: peripheral arterial occlusive disease, BMI: body mass index, UPCR: urine protein/urine creatinine ratio.
